# 905. Impact of a Real-Time Electronic Alert on Antibiotic Treatment Duration for Pneumonia in Hospitalized Patients

**DOI:** 10.1093/ofid/ofac492.750

**Published:** 2022-12-15

**Authors:** Kathleen Degnan, Steve Saw, Tiffany Lee, Vasilios Athans, Sonal Patel, Shawn Binkley, Joy Uzoma, Keith W Hamilton, David Do, Lauren Dutcher

**Affiliations:** University of Pennsylvania Perelman School of Medicine, Philadelphia, Pennsylvania; Hospital of the University of Pennsylvania, Philadelphia, Pennsylvania; Hospital of the University of Pennsylvania, Philadelphia, Pennsylvania; Hospital of the University of Pennsylvania, Philadelphia, Pennsylvania; Hospital of the University of Pennsylvania, Philadelphia, Pennsylvania; Hospital of the University of Pennsylvania, Philadelphia, Pennsylvania; Hospital of the University of Pennsylvania, Philadelphia, Pennsylvania; University of Pennsylvania Perelman School of Medicine, Philadelphia, Pennsylvania; Hospital of the University of Pennsylvania, Philadelphia, Pennsylvania; University of Pennsylvania Perelman School of Medicine, Philadelphia, Pennsylvania

## Abstract

**Background:**

Limiting antibiotic prescribing to the shortest effective duration reduces antibiotic-associated adverse events and resistance. Up to two-thirds of patients receive excessive durations of therapy for pneumonia. This study evaluated the effect of a stewardship intervention to reduce excess antibiotic duration for inpatients with pneumonia.

**Methods:**

A dashboard was developed to generate real-time alerts when inpatients at an academic medical center received antibiotics with an indication of community- or hospital-acquired pneumonia for more than 5 or 7 days, respectively. From November 2019 through April 2021, alerts were regularly reviewed by the antibiotic stewardship (AS) team and intervened upon when patients exceeded the guideline recommended duration of therapy for pneumonia without additional indications for continuing antibiotics. We compared inappropriate duration of therapy pre- and post-implementation of the dashboard by calculating the mean number of excess days of antibiotics beyond the recommended duration. Patients with SARS-CoV-2 infection and patients on hospital services that care for patients with cystic fibrosis, bronchiectasis, or immunocompromising conditions were excluded. Four other hospitals within the same health system that did not utilize the dashboard generated alerts served as a comparison group.

**Results:**

During the intervention period, the AS team reviewed 834 patients with dashboard alerts and documented 115 interventions. For alerts reviewed without intervention, reasons for lack of intervention included active Infectious Diseases consult, additional infection diagnosis requiring a longer duration, and delayed clinical improvement. In the post-implementation period there was a mean of 1.28 excess days of antibiotics for pneumonia compared to the pre-implementation mean of 1.36 excess days. In comparison, aggregating data from the hospitals not utilizing the dashboard, there was a mean of 0.67 excess days post-intervention, compared to a mean 0.62 days pre-intervention.

**Conclusion:**

The pneumonia dashboard is a potentially valuable stewardship tool which may reduce excess days of antibiotics for pneumonia. The dashboard’s impact may be improved by daily review and excluding patients with additional infection diagnoses.

**Disclosures:**

**Kathleen Degnan, MD**, Gilead: Grant/Research Support.

